# Volume Regulation and Nonosmotic Volume of Individual Human Platelets Quantified by High-Speed Scanning Ion Conductance Microscopy

**DOI:** 10.1055/a-2378-9088

**Published:** 2024-08-29

**Authors:** Konstantin Krutzke, Jan Seifert, Meinrad Gawaz, Johannes Rheinlaender, Tilman E. Schäffer

**Affiliations:** 1 Institute of Applied Physics, University of Tübingen, Tübingen, Germany; 2 Department of Internal Medicine III, Cardiology and Angiology, University of Tübingen, Tübingen, Germany

**Keywords:** hypotonic shock, platelet volume, nonosmotic fraction, platelet activation, SICM

## Abstract

**Background:**

Platelets are anucleate cells that play an important role in wound closure following vessel injury. Maintaining a constant platelet volume is critical for platelet function. For example, water-induced swelling can promote procoagulant activity and initiate thrombosis. However, techniques for measuring changes in platelet volume such as light transmittance or impedance techniques have inherent limitations as they only allow qualitative measurements or do not work on the single-cell level.

**Methods:**

Here, we introduce high-speed scanning ion conductance microscopy (HS-SICM) as a new platform for studying volume regulation mechanisms of individual platelets. We optimized HS-SICM to quantitatively image the morphology of adherent platelets as a function of time at scanning speeds up to 7 seconds per frame and with 0.1 fL precision.

**Results:**

We demonstrate that HS-SICM can quantitatively measure the rapid swelling of individual platelets after a hypotonic shock and the following regulatory volume decrease (RVD). We found that the RVD of thrombin-, ADP-, and collagen-activated platelets was significantly reduced compared with nonactivated platelets. Applying the Boyle–van't Hoff relationship allowed us to extract the nonosmotic volume and volume fraction on a single-platelet level. Activation by thrombin or ADP, but not by collagen, resulted in a decrease of the nonosmotic volume, likely due to a release reaction, leaving the total volume unaffected.

**Conclusion:**

This work shows that HS-SICM is a versatile tool for resolving rapid morphological changes and volume dynamics of adherent living platelets.

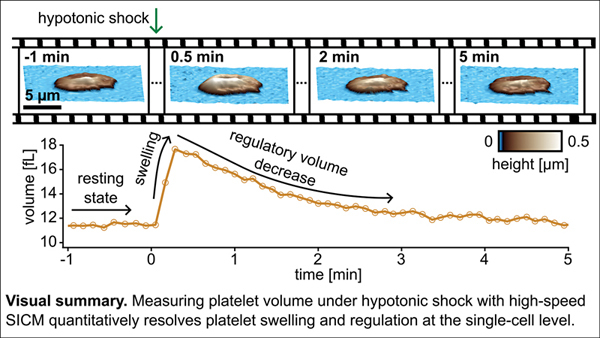

## Introduction

Platelets are one of the three main cellular components present in mammalian blood and play a pivotal role in human physiology, particularly in hemostasis and the intricate process of wound closure. After vessel injury, platelets are activated, adhere to extracellular matrix components such as collagen or fibrinogen, and release chemical signals to attract more platelets to the site of injury. 1 2 Platelets thereby undergo a shape change to close the injured vessel by agglomeration and formation of a blood clot. 3 4 Activation can be triggered by various factors, 4 5 for example, by mechanical or enzymatic stimulation, 6 7 and uncontrolled platelet activation increases the risk of thrombosis or stroke. 8 9

Maintaining a constant platelet volume is critical for correct platelet function. 10 For example, it was shown that water-induced swelling of platelets might promote their activity and thereby initiate thrombosis. 11 Therefore, platelets have established an effective volume regulation mechanism (regulatory volume decrease, RVD), 12 based on the passive transport of water by water channels (aquaporins). 11 The regulation of platelet volume is associated with a change in the osmolarity of the surrounding medium, which can be an important factor, for example, in blood transfusion. 13 14 When exposed to a rapid osmolarity change, the platelet changes its volume in response to the osmolarity change of the medium to adapt to the extracellular environment, mediated by an influx or efflux of water through aquaporins in the cell membrane into or out of the cell. 11 12 15 However, despite its potential significance, the impact of platelet activation on RVD and its role in physiological and pathological processes has not been widely investigated. 11 16 Single-cell measurements present a significant advantage in this context, as they allow for the characterization of individual platelets, thereby capturing the substantial variance that exists within platelet populations.

Methods to quantify platelet volume such as light transmittance or impedance measurements are limited as they only measure volume qualitatively or only determine the average volume of a platelet population. On the other hand, single-platelet imaging techniques such as atomic force microscopy 6 17 or fluorescence microscopy 18 19 might induce unintended platelet activation or often require fixation or fluorescent labeling of the platelets. 17 20

We therefore applied high-speed scanning ion conductance microscopy 21 22 (HS-SICM) to record topography image sequences of individual platelets to investigate the dynamics of water-induced swelling and subsequent volume regulation. 7 23 As HS-SICM can image live cells with submicrometer resolution without mechanical contact, it is ideal for quantitative assessment of platelet shape and volume 24 without the risk of platelet activation. 7 25 For example, SICM has recently been used to study platelet morphology, 26 mechanics, 7 migration, 25 and thrombus formation. 27 By optimizing the scanning speed and pixel resolution to measure the volume of individual platelets, we observe rapid volume changes in swelling platelets with a temporal resolution of 7 seconds per frame and a precision of 0.1 fL. We demonstrate that the peak volume and the swelling rate after hypotonic shock directly depend on the osmolarity of the extracellular medium. RVD was suppressed when platelets were investigated in the presence of thrombin, adenosine diphosphate (ADP), or collagen. We also showed that the nonosmotic volume in platelets was reduced in thrombin- or ADP-treated, but not in collagen-treated platelets.

## Methods

### Human Platelet Isolation and Preparation

All procedures were approved by the institutional ethics committee (273/2018BO2) and comply with the Declaration of Helsinki. Freshly drawn venous blood of healthy volunteers was used for platelet isolation by using monovettes filled with anticoagulant acid citrate dextrose (at a ratio of 1:4) (04.1926.001, Sarstedt, Nümbrecht, Germany). After centrifugation at 200× *g* for 20 minutes, platelet-rich plasma was collected and transferred into Tyrode-HEPES buffer solution (136.89 mM NaCl, 2.81 mM KCl, 11.9 mM NaHCO _3_ , 1.05 mM MgCl _2_ , 0.42 mM NaH _2_ PO _4_ , 5.56 mM D-glucose, 1 g/L bovine serum albumin, 4 mM HEPES), pH 6.5, at a ratio of 1:3. After centrifugation at 880× *g* for 10 minutes, platelets were carefully resuspended in 1 mL Tyrode-HEPES buffer solution, pH 7.4. The Tyrode-HEPES buffer solution had a standard osmolarity of 295 mOsmol/L and is referred to as “isotonic” in the following.

For HS-SICM measurements, washed platelets were added to a cell culture dish (627160, Greiner Bio-One GmbH, Kremsmünster, Austria). After 10 seconds, nonadherent platelets were removed by carefully washing three times with isotonic Tyrode-HEPES buffer solution, pH 7.4, and adherent platelets were allowed to spread for 10 minutes. Afterwards, the dish was mounted in the HS-SICM setup and HS-SICM measurements were performed at room temperature.

To inhibit the RVD, washed platelets were imaged in isotonic Tyrode-HEPES buffer solution with an increased KCl (+45 mM) and a decreased NaCl (−45 mM) concentration (unchanged osmolarity; this buffer solution is referred to as high K ^+^ buffer and respective platelets as high K ^+^ -treated in the following). 12

To determine the influence of the actin cytoskeleton on the swelling and volume regulation behavior, 50 µM cytochalasin D (cytoD; Cay11330–5, Cayman Chemical Company, Michigan, United States, solved in DMSO) was added to the cell culture dish with adherent platelets during HS-SICM imaging. The solvent DMSO did not affect the platelet volume ( Supplementary Fig. S4D , available in the online version).

Activation of platelets was performed by incubating platelets in suspension for 30 seconds with 0.1 U/mL thrombin (T6884–250UN, Sigma-Aldrich, St. Louis, Missouri, United States), or 10 µM ADP (Sigma-Aldrich) before adhesion and spreading. 28 29 30 Thrombin or ADP was present at the given concentration during the whole measurement. For platelet activation with collagen, 31 32 the cell culture dish was incubated with 0.1 mg/mL collagen (Collagen Reagens HORM, Takeda Pharma GmbH, Vienna, Austria) for 1 hour at 37°C. After incubation, the dish was washed three times with Tyrode-HEPES buffer solution, pH 7.4.

A commercial cell counter (impedance measurement principle, Sysmex KX-21N, Sysmex Corporation, Kobe, Japan) was used for the determination of the mean platelet volume (MPV) and the platelet distribution width (PDW) of platelets in whole blood.

### HS-SICM Imaging

**Fig. 1:**
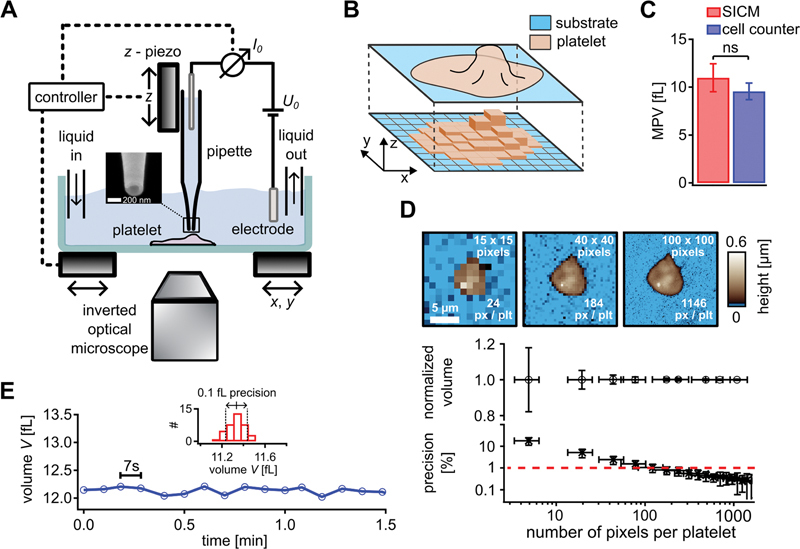
Platelet topography imaging and volume measurement with HS-SICM. ( **A** ) Schematic of the HS-SICM setup using a nanopipette (inset shows scanning electron microscopy image, pipette inner opening radius 90 nm) that is moved relative to the sample in the *x* -, *y* -, and *z* -directions by piezo scanners. A bias voltage *U*_0_ applied between two electrodes induces a distance-dependent ion current *I*_0_ (also see Supplementary Fig. S1A , available in the online version). The sample buffer solution can be exchanged by using an inlet and an outlet to induce an osmotic shock. ( **B** ) Schematic for the calculation of platelet volume from the pixels of a topography image. ( **C** ) Average MPV of platelets measured with HS-SICM and a cell counter. ( **D** ) HS-SICM topography images (top row) with different pixel resolutions (15 × 15, 40 × 40, and 100 × 100 pixels, resulting in 24, 184, and 1,146 pixels per platelet). Normalized volume (middle row) and volume measurement precision (bottom row) averaged over down-sampled platelet images for *N* = 26 platelets versus number of pixels per platelet. A volume measurement precision of 1% (red dashed line) corresponds to approximately 200 pixels per platelet. Error bars show the standard deviation for normalized volume (middle row) and SESD for measurement precision (bottom row). ( **E** ) Volume measured for one fixed platelet over time with HS-SICM at 7 seconds per frame. The histogram (inset) shows the volume distribution giving 0.1 fL precision (standard deviation). HS-SICM, high-speed scanning ion conductance microscopy; MPV, mean platelet volume; SESD, standard error of the standard deviation.

We used a self-built HS-SICM setup with an electrolyte-filled nanopipette ( Fig. 1A ). Nanopipettes with inner opening radii of 80 to 100 nm ( Fig. 1A , validated by scanning electron microscopy) were manufactured from borosilicate glass capillaries (Kwik-Fill glass capillaries, World Precision Instruments Inc., Florida, United States) using a CO _2_ -laser-based pipette puller (P2000, Sutter Instruments, California, United States). The principle of HS-SICM is described elsewhere in detail. 21 25 33 34 In brief, a voltage of 250 mV, applied between two Ag/AgCl electrodes, one outside and one inside the pipette, induces an ion current that is dependent on the pipette–surface distance. The three-dimensional topography of the sample surface is acquired by using the ion current as a feedback signal and scanning the pipette over the sample using piezo actuators. The HS-SICM setup was operated in hopping/backstep mode 35 with an approach speed of 250 µm/s, an ion current trigger of 99.5% of the saturation current for the retraction of the pipette ( Supplementary Fig. S1A , available in the online version, dashed lines), and a retract distance of 0.5 µm. The vertical pipette position at the trigger event was stored as the sample height at that location.

To increase the time resolution of HS-SICM imaging, we used masks for scanning only relevant regions (platelet area dilated by 2 µm and 20% random coverage of the substrate area; Supplementary Fig. S1E , available in the online version). With 40 × 40 pixels per frame and a standard scan size between 15 and 20 µm, the typical acquisition time was 7 seconds per frame ( Fig. 1E ).

For obtaining the MPV and the PDW using HS-SICM, the topography of adherent platelets was imaged using 100 × 100 µm ^2^ scans with a pixel resolution of 150 × 150 pixels.

### Application of Osmotic Shock

To induce a hypotonic shock, the isotonic Tyrode-HEPES buffer solution (isotonic osmolarity of 295 mOsmol/L) was quickly exchanged with hypotonic buffer solution (Tyrode-HEPES buffer solution diluted with pure H _2_ O [HPLC quality, Fischer Chemical GmbH, Schwerte, Germany] at different volume percentages (10, 20, 30, 40, or 50% pure H _2_ O of the final solution]), leading to a rapid decrease in osmolarity (265, 236, 206, 177, or 147 mOsmol/L). To induce a hypertonic shock, the isotonic Tyrode-HEPES buffer solution was quickly exchanged with hypertonic buffer solution (Tyrode-HEPES buffer mixed with D-sorbitol [S1876–100G, Sigma Aldrich, Missouri, United States] at different concentrations [59, 118, or 154 mM]), leading to a rapid increase in osmolarity (354, 413, or 447 mOsmol/L) while not affecting platelet viability. 36

For normal (untreated) platelets, platelets treated with high K ^+^ buffer solution, and thrombin-treated platelets, HS-SICM measurements were started 5 minutes before the osmotic shock was induced. For cytoD measurements, adherent platelets were treated with cytoD for 10 minutes before the hypotonic shock was induced (no change in cytoD concentration) and HS-SICM measurements were started 5 minutes before the treatment with cytoD ( Supplementary Fig. S4C , available in the online version).

For solution exchange, a 3D-printed (Formlabs 3, Somerville, Massachusetts, United States) cell culture dish holder with two connections, one for extracting the current buffer solution, and one for adding the exchange buffer solution, was used. HS-SICM imaging was maintained during solution exchange. Although changing the salt concentration of the electrolyte to introduce a hypotonic shock led to a decrease in ion concentration and thus to a decrease in measured ion current ( Supplementary Fig. S1F , available in the online version), the quality of HS-SICM images was almost unaffected. In the case of hypertonic shock, no decrease in image quality was observed.

### HS-SICM Data Analysis

HS-SICM topography images of platelets were processed and analyzed using Igor Pro 9 (WaveMetrics, Inc., Portland, Oregon, United States). Platelets were identified by a pixel height threshold of *h* = 50 nm, and pixels below this threshold were considered as substrate. All topography images were corrected for tilt and z-offset by first-order line flattening. 37 To calculate the area *A* of each platelet, the sum of all pixel areas corresponding to the platelet was formed,  . The platelet volume *V* was calculated by multiplying the area *A* with the mean height  of the platelet,  , which is mathematically equivalent to summing up the volumes of all pixels corresponding to the platelet ( Fig. 1B ). 24

For quantifying the influence of the pixel resolution on the measured platelet volume, high-resolution images (giving the “true” volume) were downscaled with systematically varying pipette position offsets. Afterwards, the platelet volumes for the downscaled and flattened images were calculated.

The MPV was calculated as the arithmetic mean of the platelet volumes measured by HS-SICM. The PDW was measured as the width at 20% of the maximum value from log-normal distribution fits ( Supplementary Fig. S1C, D , available in the online version).

For investigating the precision of the volume measurement of our HS-SICM system ( Fig. 1E ), platelets were fixed in 2% formaldehyde for 10 minutes at room temperature. Then, *N* = 5 fixed platelets were imaged over time (at least 20 minutes).

**Fig. 2:**
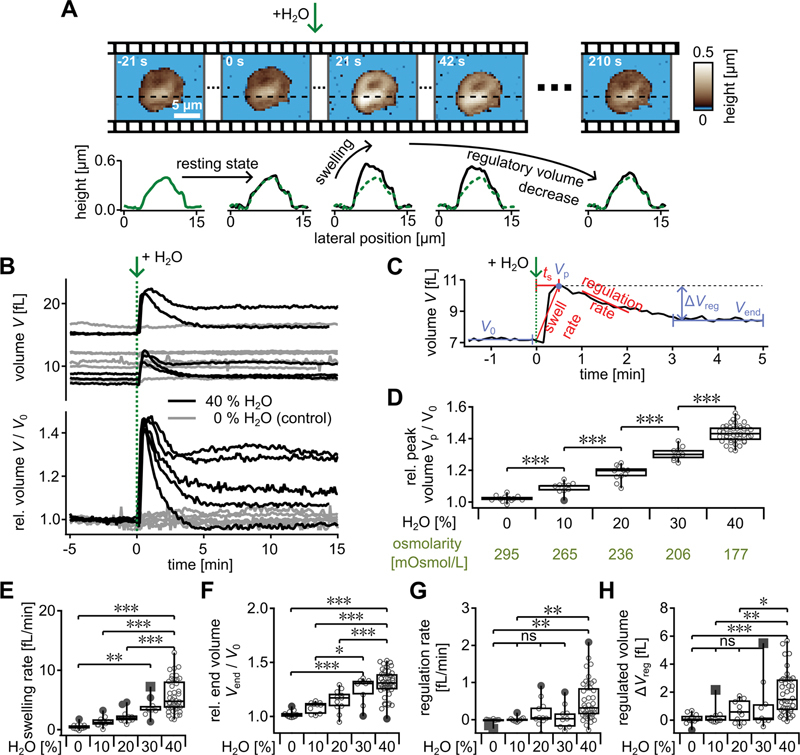
Quantitative single-platelet volume measurements with HS-SICM, resolving rapid volume dynamics of living platelets after hypotonic shock. ( **A** ) Sequence of topography images (top) and corresponding height profiles (bottom). The dashed line corresponds to the platelet before exposure to a hypotonic shock of 40% H _2_ O at *t* = 0 s. Every third image is shown. The complete image sequence is shown in Supplementary Fig. S2A (available in the online version) and in Video 1 (available in the online version). The hypotonic shock induces a rapid height increase (swelling) of the platelet, followed by a slower height decrease (regulatory volume decrease). ( **B** ) Time dynamics of the volume (top) and the volume relative to the initial volume *V*_0_ (bottom) of individual platelets exposed to a hypotonic shock at *t* = 0 with 40% H _2_ O (black curves) or 0% H _2_ O (gray curves, control). ( **C** ) Representative volume vs. time curve showing the definitions of the initial volume *V*_0_ , the peak volume ( *V*_p_ ), the regulated volume (Δ *V*_reg_ ), the final volume ( *V*_end_ ), the swelling time *t*_s_ , the swelling rate, and the regulation rate. ( **D** ) Relative peak volume *V*_p_ / *V*_0_ after a hypotonic shock with varying osmolarity. ( **E** ) Swelling rate, ( **F** ) relative end volume *V*_end_ / *V*_0_ , and ( **G** ) regulation rate for a hypotonic shock with varying osmolarity. ( **H** ) Regulated volume Δ *V*_reg_ of platelets after a hypotonic shock with varying osmolarity. Number of platelets (D–H): *N* = 12 (0% H _2_ O), 10 (10%), 11 (10%), 9 (30%), and 42 (40%). HS-SICM, high-speed scanning ion conductance microscopy.

For all time-dependent measurements, *t* = 0 was defined as the time of the hypotonic shock. The time interval before the hypotonic shock [−5 minutes; 0 minute] was used for the calculation of the initial volume *V*_0_ (average volume before the hypotonic shock). The relative volume was then calculated as *V* / *V*_0_ with the initial volume *V*_0_ ( Fig. 2C ). The swelling time *t*_s_ ( Fig. 2C ) was determined by calculating the time between the hypotonic shock (green arrow at *t* = 0) and the peak volume *V*_p_ . The platelet swelling rate was defined as  . The regulation rate was determined as the slope of the volume between *t* = 1 and 2 min after the peak volume *V*_p_ by applying a line fit ( Fig. 2C ). The end volume *V*_end_ was defined as the average volume between *t* = 3 min and 5 min after the hypotonic shock. The regulated volume was calculated as Δ *V*_reg_ = *V*_p_ – *V*_end_ . Hence, for Δ *V*_reg_ ∼ 0, a platelet was interpreted as nonregulating.

The platelet-to-platelet variation of the RVD was calculated as the standard deviation σ of the relative end volume *V*_end_ / *V*_0_ with standard error of the standard deviation (SESD =  , with number of platelets *N* ).

To determine the nonosmotic fraction of the platelets, we used the Boyle–van't Hoff relationship 38 for cells under osmotic conditions, which assumes a linear correlation between the (relative) cell volume and the inverse tonicity 12 39 Ψ (here: ratio of the isotonic osmolarity and the hypo-/hypertonic osmolarity). For individual platelets, two data points were available: *V*_0_ / *V*_0_ = 1 at Ψ = 1 before the osmotic shock, and *V*_p_ / *V*_0_ at the given Ψ after the osmotic shock (before regulation). We plotted the relative platelet volumes as a function of Ψ and applied a line fit to the data points. The value of the fit at Ψ = 0 gives the osmotically inactive, nonosmotic fraction of the platelets. 12 For the calculation of the nonosmotic fraction of the platelet population, the line fit was applied to the averaged volumes at the given Ψ.

### Statistical Analysis

Statistical tests were performed using Igor Pro 9 (WaveMetrics, Inc., Portland, Oregon, United States). Box plots show the median and the lower and upper quartiles. For the comparison between two groups, Student's *t* -test was used. For comparison between three or more groups, Tukey's range test was used. Variances were tested with the F-test for significance. The results were considered as significantly different for *p* ≤0.05 (*), *p* ≤0.01 (**), and *p* ≤0.001 (***), and as not significantly different (ns) for *p* >0.05. Platelets from three to eight independent donors were measured.

## Results and Discussion

### Optimization of Fast Volume Measurements with HS-SICM

The topography of individual adherent, spread platelets was imaged using HS-SICM ( Fig. 1A ). Platelets usually had a spread-out morphology with a flat lamellipodium at the outer regions and a higher platelet body at the center ( Supplementary Fig. S1B , available in the online version). In a topography image, the platelet volume corresponds to the sum of the effective volume of all pixels of the platelet ( Fig. 1B ). To verify that SICM accurately measures platelet volume, we compared the MPV of three different donors as obtained from SICM topography images with the MPV measured with a conventional cell counter as “gold standard,” revealing no significant difference ( Fig. 1C and Supplementary Fig. S1C [available in the online version]). However, within individual donors, there was a trend for slightly larger MPV and PDW with SICM compared with the cell counter ( Supplementary Fig. S1D , available in the online version). This discrepancy could likely arise from the difference in measurement conditions, since we measured adherent washed platelets with SICM as opposed to platelets from whole blood in solution with the cell counter.

The measurement speed of SICM is dependent on numerous factors and the acquisition of a topography image typically takes several minutes, 35 limiting the ability to measure rapid morphological changes of individual human platelets. The acquisition time in HS-SICM in the hopping mode is proportional to the number of pixels of the image. At a low number of pixels per image, the acquisition time is short, but the image resolution is low due to a high degree of pixelation of the platelet ( Fig. 1D , left image), making volume measurements less precise. To find the optimum number of pixels per platelet for a given volume precision, we recorded a high-resolution image (200 × 200 pixels) of a platelet and progressively down-sampled this image to generate lower resolution images (100 × 100, 40 × 40, and 15 × 15 pixels; Fig. 1D , top; also see the Methods section). From each of these images, we measured the platelet volume (normalized by the “true” platelet volume from the respective high-resolution image). Repeating this process for 26 images of different platelets allowed us to plot the average of the measured normalized volumes and the average precision of the volume measurements as a function of the number of pixels per platelet ( Fig. 1D , center and bottom). While the average normalized volume stayed constant for an increasing number of pixels per platelet ( Fig. 1D , center), the precision of that measurement improved ( Fig. 1D , bottom). For a volume measurement precision of 1%, we need about *n* = 200 pixels per platelet ( Fig. 1D , bottom, red dashed line). For a typical platelet area *A* , this corresponds to a required pixel size of  . Assuming a circular platelet of diameter *d* , the required pixel size is  , which amounts to *d* / *s*_px_ = 16 pixels across the platelet. For a typical platelet diameter of 7 µm ( Supplementary Fig. S1B , available in the online version), the required pixel size is *s*_px_ = 440 nm (for 1% precision in the platelet volume measurement). In our experiments, we therefore chose an image resolution of 40 × 40 pixels with a scan size of 15 to 20 µm.

To further speed up the imaging, we reduced the number of pixels acquired on the substrate around the platelet by computing a dynamic mask around the platelet ( Supplementary Fig. S1E , available in the online version, images). These optimizations facilitated a reduction in acquisition time by a factor of approximately 2.5 in this example ( Supplementary Fig. S1E , available in the online version, right). As a result, we were able to capture rapid topography and volume changes of individual living platelets with acquisition times of down to 7 seconds per frame ( Fig. 1E and Supplementary Fig. S2A [available in the online version]) (comparable with values reported in other HS-SICM publications 40 41 ) and with a precision of typically 0.1 fL ( Fig. 1E , inset) (which reflects the 1% precision from the pixelation set above).

### Platelet Swelling after Hypotonic Shock

In contrast to previous studies, which measured suspended platelet volume changes in solution, averaged over a whole population of platelets, or performed only qualitative measurements, 14 15 42 our approach with HS-SICM allows capturing quantitative, time-lapse volume changes of adherent platelets undergoing hypotonic shock. Platelets showed a rapid swelling with an increase in platelet height after hypotonic shock ( Fig. 2A , Supplementary Fig. S2A [available in the online version], and Video 1 [available in the online version]). Subsequently, the height slowly decreased again toward almost the initial height (RVD; Fig. 2A ). From such image sequences, we measured the platelet volume *V* ( Fig. 2B , top) and the volume relative to the initial volume *V*_0_ ( Fig. 2B , bottom) as a function of time. The volume of platelets exposed to a hypotonic shock of 40% H _2_ O rapidly increased within seconds after the shock ( Fig. 2B , black curves). Within ≈1 minute after the hypotonic shock, the volume started to decrease again and became constant at ≈5 minutes. Platelets without a hypotonic shock did not show any significant increase in volume ( Fig. 2B , gray curves). Platelets during hypotonic shock with lower H _2_ O percentages generally showed a weaker volume change but a similar time behavior ( Supplementary Fig. S2B–D , available in the online version). These volume dynamics can be parameterized ( Fig. 2C ) as the initial volume *V*_0_ before the hypotonic shock, the peak volume *V*_p_ reached at the swelling time *t*_s_ after the hypotonic shock, the end volume *V*_end_ , the regulated volume Δ *V*_reg_ *= V*_p_ *− V*_end_ , the swelling rate, and the regulation rate.

The relative peak volume *V*_p_ / *V*_0_ increased significantly for an increasing H _2_ O percentage and hence for decreasing osmolarity ( Figs. 2D and Supplementary Fig. S2B–D [available in the online version]). The swelling rate also increased with the H _2_ O percentage ( Fig. 2E ). In contrast, the swelling time did not depend on the H _2_ O percentage and was therefore independent of the osmolarity ( Supplementary Fig. S3A , available in the online version). This indicates that a hypotonic shock with a larger osmotic change induces a larger water influx into the platelet, which supports the assumption that the volume increase in platelets is driven by diffusion. 43 44 This assumption was further upheld by the roughly linear correlation ( *r* = 0.66 ± 0.091, *p* = 3.04∙10 ^−10^ ) between the initial platelet volume *V*_0_ (as a measure of platelet size) and the swelling rate ( Supplementary Fig. S3B , available in the online version), showing that larger platelets with a larger surface area can take up larger amounts of H _2_ O per time. Consequently, the swelling rate of platelets showed a significant size-dependency for a hypotonic shock with 40% H _2_ O when comparing small ( *V*_0_ < 12 fL) and large ( *V*_0_ ≥ 12 fL) platelets ( Supplementary Fig. S3C , available in the online version, left). In contrast, the regulation rate did not depend on platelet size ( Supplementary Fig. S3C , available in the online version, right). For better comparison of the end volume *V*_end_ (volume plateau after regulation) between differently sized platelets and different H _2_ O percentages, we investigated the relative end volume *V*_end_ / *V*_0_ , showing a significant increase with H _2_ O percentage ( Fig. 2F ).

The regulation rate was significantly increased ( *p* = 0.0012, compared with control) only after hypotonic shock with 40% H _2_ O ( Fig. 2G ). Thus, both the regulated volume Δ *V*_reg_ ( Fig. 2H ) and the relative regulated volume Δ *V*_reg_ / *V*_0_ ( Supplementary Fig. S3D , available in the online version) were significantly ( *p* = 0.00098, *p* = 0.00012, respectively, compared with control) increased only after a hypotonic shock with 40% H _2_ O and were much smaller for 30% H _2_ O or lower, indicating that RVD is more pronounced at a hypotonic shock with lower osmolarity. 45 In the following, we therefore used 40% H _2_ O to induce a hypotonic shock. For comparison, platelets exposed to hypertonic shock rapidly shrank but did not show a notable regulatory volume increase (RVI; Supplementary Fig. S2E , available in the online version). This aligns with previous findings that platelets, among other cell types, respond to hypertonic shrinking with activation of Na ^+^ /H ^+^ exchange but do not exhibit a detectable RVI. 12 This behavior may be explained by different ion channels involved in RVD and RVI. In RVD, mainly K ^+^ and Cl ^−^ are secreted, 46 leading to an efficient change of the intracellular osmolarity by water efflux, whereas in RVI, mainly the Na ^+^ /H ^+^ exchanger is activated, which might decrease the net gain of osmolytes in RVI. 47 However, some cell types can exhibit an effective RVI after hypertonic shock, but the reason for the different RVI behaviors is yet unknown. 48

The swelling induced by the hypotonic shock primarily caused an increase in platelet height, while the platelet area did not significantly change ( Supplementary Fig. S3E , available in the online version). Interestingly, for a hypotonic shock with 80% H _2_ O, which led to an average relative peak volume of *V*_p_ / *V*_0_ = 2.32, the formation of a filopodium could be observed within minutes after the hypotonic shock ( Supplementary Fig. S3F, G [available in the online version]; also see Videos 2 and 3 [available in the online version]), which is a possible indicator of platelet apoptosis. 6 The absence of filopodia at 40% H _2_ O and below supports the assumption that platelet viability is not notably decreased by hypotonic shocks with lower osmolarities and that platelets can swell and regulate their volume without apoptosis. 12

### RVD of Platelets Is Suppressed after Platelet Activation

**Fig. 3:**
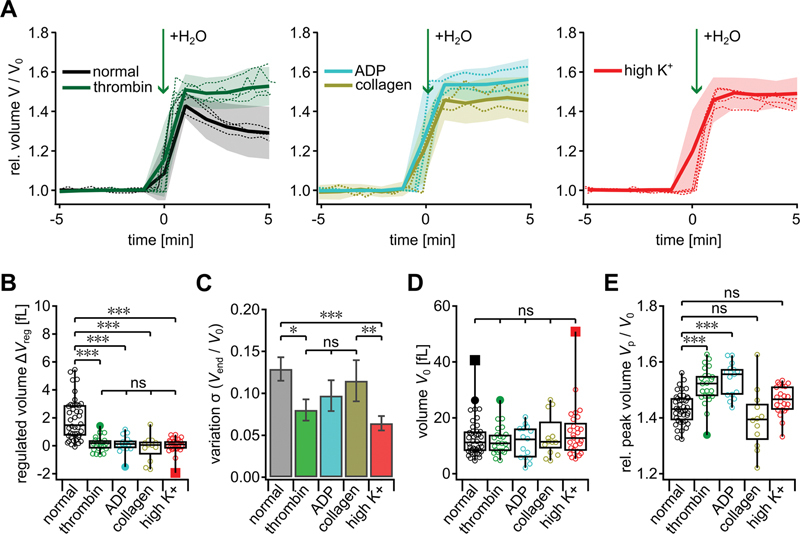
RVD of individual platelets after a hypotonic shock with 40% H _2_ O. ( **A** ) Relative volume vs. time of representative individual platelets (dashed lines) during a hypotonic shock for different platelet treatments. Solid lines and shaded areas represent the mean and standard deviation, respectively, of all measured platelets. ( **B** ) Regulated volume Δ *V*_reg_ for normal (untreated), thrombin-treated, ADP-treated, collagen-treated, and high K ^+^ -treated platelets. ( **C** ) Variation σ of the relative end volume *V*_end_ / *V*_0_ . Error bars show the SESD. ( **D** ) Initial volume *V*_0_ and ( **E** ) relative peak volume *V*_p_ / *V*_0_ for different platelet treatments. Significance in (C) was determined using the F-test. Number of cells (B–E): *N* = 43 (normal), 23 (thrombin), 15 (ADP), 12 (collagen), and 28 (high K ^+^ ). SESD, standard error of the standard deviation; RVD, regulatory volume decrease.

To investigate the influence of platelet activation on the RVD, we first used thrombin as a potent platelet activator. 7 23 While normal (untreated) platelets showed a normal RVD after a hypotonic shock with 40% H _2_ O ( Fig. 3A , left, black traces), as already reported above, thrombin-treated platelets showed no notable RVD after the hypotonic shock, but remained at a constantly high volume ( Fig. 3A , left, green traces). Consequently, the regulated volume Δ *V*_reg_ of thrombin-treated platelets was significantly reduced ( *p* = 1.89∙10 ^−8^ ) compared with normal platelets ( Fig. 3B ).

Taking advantage of the single-platelet resolution of HS-SICM, we calculated the platelet-to-platelet variation σ of the RVD as a function of time ( Fig. 3A , shaded areas) and the standard deviation σ of the relative end volume *V*_end_ / *V*_0_ ( Fig. 3C ). For thrombin-treated platelets, the platelet-to-platelet variation σ was significantly reduced ( *p* = 0.019) compared with normal platelets, which indicates a more homogenous behavior of the platelets after hypotonic shock.

To investigate whether activation generally suppresses RVD in platelets, we further used ADP 49 50 and collagen 32 ( Supplementary Fig. S4A , available in the online version) on platelets undergoing hypotonic shock ( Fig. 3A , center, light blue and yellow traces). Like thrombin-treated platelets, ADP and collagen both suppressed RVD after hypotonic shock with 40% H _2_ O as indicated by a constant high relative volume after hypotonic shock ( Fig. 3A ). Consequently, the regulated volume Δ *V*_reg_ was significantly ( *p* = 2.75∙10 ^−5^ and *p* = 7.88∙10 ^−6^ , respectively) reduced compared with normal platelets ( Fig. 3B ). Further, the platelet-to-platelet variation σ of the RVD for ADP- and collagen-treated platelets was also smaller and similar to thrombin-treated platelets ( Fig. 3C ). Therefore, we propose that activation inhibits RVD in adherent platelets.

For comparison, we measured platelets in the presence of a high K ^+^ buffer, which is known to effectively inhibit the RVD. 12 As expected, platelets exposed to high K ^+^ buffer did not show a strong RVD ( Fig. 3A , right, red curves) and their regulated volume Δ *V*_reg_ was close to zero, significantly smaller ( *p* = 4.24∙10 ^−10^ ) than for normal platelets and similar to activated platelets ( Fig. 3B ). Importantly, the platelet-to-platelet variation was also smaller than for normal platelets ( *p* = 0.00028). During RVD, the efflux of K ^+^ and Cl ^−^ ions leads to an efflux of water and a decrease in cell volume, 36 51 which is in line with the reduced efficiency of RVD in the presence of high K ^+^ solutions. 12 52 As the corresponding ion channels are also involved in platelet activation, 46 53 treatment with thrombin, ADP, or collagen could possibly reduce the ability of the ion channels in mediating RVD. Additionally, the absence of RVD in activated platelets may be further attributed to a change in the actin cytoskeleton and in ion channel and transporter activity. 12 54 55 56 57

As activation is known to affect the platelet cytoskeleton, we also investigated a possible influence of the cytoskeleton on the RVD, as it is known for many cell types. 58 59 We therefore treated adherent platelets with 50 µM cytoD during HS-SICM imaging to inhibit actin polymerization ( Supplementary Fig. S4B , available in the online version). 58 60 This led to a slight volume increase ( Supplementary Fig. S4C , available in the online version), indicating reduced internal stress from active actin cytoskeleton remodeling during adhesion and spreading. 61 62 63 Despite this, the RVD was still present and the regulated volume Δ *V*_reg_ , the platelet-to-platelet variation σ, the initial volume *V*_0_ , and the relative peak volume *V*_p_ / *V*_0_ after hypotonic shock with 40% H _2_ O were similar to normal platelets ( Supplementary Fig. S4E–H , available in the online version). Therefore, RVD in platelets appears to be independent of the actin cytoskeleton, unlike for other cell types, where an intact actin cytoskeleton is crucial for effective RVD. 58 59 64

### The Nonosmotic Fraction of Platelets Varies Depending on the Activation Agonist

The initial platelet volume *V*_0_ was identical for all treatments ( Fig. 3D ). The relative peak volume *V*_p_ / *V*_0_ was significantly different for platelets treated with thrombin or ADP ( *p* = 0.00013, and *p* = 2.75∙10 ^−5^ , respectively; Fig. 3E ). In contrast, collagen-treated platelets had a similar relative peak volume *V*_p_ / *V*_0_ to normal platelets ( Fig. 3E ).

**Fig. 4:**
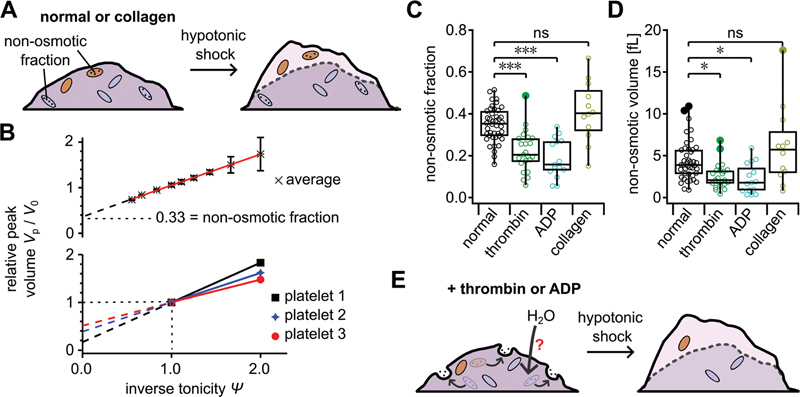
Determination of the nonosmotic fraction of swelling platelets with HS-SICM. ( **A** ) Schematic of the behavior of the nonosmotic fraction during hypotonic shock of normal or collagen-treated platelets. ( **B** ) Relative peak volume *V*_p_ / *V*_0_ versus the inverse tonicity Ψ averaged for many normal platelets (top) and for three individual normal platelets (bottom). Following the Boyle–van't Hoff relationship, the osmotically inactive, nonosmotic volume fraction is given by the *y* -intercept of the line. ( **C** ) Nonosmotic fraction and ( **D** ) nonosmotic volume of normal, thrombin-, ADP-, and collagen-treated platelets. ( **E** ) Schematic of the possible action of thrombin or ADP on a platelet before and after hypotonic shock. The nonosmotic volume is denoted by the colored objects inside the platelet. Thrombin- and ADP-induced vesical fusion with the platelet membrane is indicated by small arrows. Number of cells (C, D): *N* = 82 (B); *N* = 42 (normal), 23 (thrombin), 15 (ADP), and 12 (collagen). HS-SICM, high-speed scanning ion conductance microscopy.

This observation prompted us to investigate the nonosmotic volume of platelets. Similar to other mammalian cells, the cytoplasm of platelets contains both osmotically active and inactive components 43 65 ( Fig. 4A ). Plotting the average relative peak volume *V*_p_ / *V*_0_ versus the inverse tonicity ( Fig. 4B , top) and using the Boyle–van't Hoff relationship 38 for cells under different osmotic conditions (see the Methods section) allowed us to extract the nonosmotic fraction as the intercept of the extrapolated line fit with the *y* -axis: 0.33 ± 0.01. This value is consistent with the literature for platelets 12 and other cells. 66 Moreover, we were able to determine the nonosmotic fraction of individual platelets ( Fig. 4B , bottom, intercepts with the *y* -axis). We observed a significant variation in the nonosmotic fraction among individual platelets, ranging between 0.2 and 0.5 ( Fig. 4C , normal) and between 1 and 12 fL ( Fig. 4D , normal). The nonosmotic volume was found to be proportional to the initial volume *V*_0_ ( Supplementary Fig. S5A , available in the online version). Consequently, the nonosmotic fraction was independent of the initial volume *V*_0_ ( Supplementary Fig. S5B , available in the online version).

For thrombin- or ADP-induced activation, both the nonosmotic fraction ( *p* = 1.4236∙10 ^−5^ , *p* = 2.2345∙10 ^−6^ , respectively) and the nonosmotic volume ( *p* = 0.0319, *p* = 0.0274, respectively) were significantly decreased by approximately 42% (thrombin) and 55% (ADP) compared with normal platelets ( Fig. 4C, D ). For collagen-induced activation, in contrast, the nonosmotic fraction and volume were unchanged ( Fig. 4A, C, D ). These observations are consistent with previous studies that have demonstrated that thrombin 67 68 and ADP 28 30 induce a “release reaction” in platelets, causing various soluble and nonsoluble components, for example, granule fractions, serotonin, or platelet factor 4, to be released into the extracellular space, while collagen activation prompts the release of mostly soluble compounds. 31 Thrombin and ADP trigger the release of Ca ^2+^ into the cytosol, promoting vesicle fusion with the membrane ( Fig. 4E , small arrows). 69 70 The extracellular release of Ca ^2+^ is typically mediated by the Na ^+^ /Ca ^2+^ exchanger, which is activated as a secondary response to elevated intracellular Ca ^2+^ levels. 11 53 71

As the measured initial volume *V*_0_ was similar for normal, thrombin- and ADP-treated platelets ( Fig. 3D ), we hypothesize that the release of nonosmotic volume 72 into the extracellular space by vesicle fusion is accompanied by an influx of water into the platelet to maintain the initial volume ( Fig. 4E ). A possible explanation for this phenomenon could be the relief of membrane tension by exocytotic vesicle fusion, which could trigger water influx ( Fig. 4E , large arrow). 73

## Conclusion



**Video 1** Image sequence of platelet topography using HS-SICM with an acquisition time of 7 s per frame. A hypotonic shock with 40% H _2_ O was induced at *t* = 5 min.


**Video 2** Image sequence of platelet topography using HS-SICM with an acquisition time of 7 s per frame. A hypotonic shock with 80% H _2_ O was induced at *t* = 5 min, leading to the formation of a filopodium.


**Video 3** Image sequence of platelet topography using HS-SICM with an acquisition time of 7 s per frame. A hypotonic shock with 80% H _2_ O was induced at *t* = 5 min, leading to the formation of a filopodium.


We investigated dynamic volume changes of individual human platelets by topography imaging with HS-SICM. This technique permits noninvasive imaging of living cells at submicrometer spatial resolution and a time resolution on the second scale without mechanical interaction with the sample. We optimized the time resolution of HS-SICM imaging for precise volume measurement, linking a threshold of 200 pixels per platelet to a measurement precision of 1% ( Fig. 1D ). The imaging speed was further increased by reducing the number of pixels in the background, leading to an acquisition time of 7 seconds per frame and a volume measurement precision of 0.1 fL ( Fig. 1E ).

We used our HS-SICM setup to investigate the behavior of adherent and spread platelets in response to a hypotonic shock ( Fig. 2 ). The platelet volume rapidly increased after hypotonic shock and was slowly regulated back toward its initial value. The relative peak volume of platelets showed a significant volume increase with an increase in H _2_ O percentage, and hence a decrease in osmolarity. Moreover, the swelling rate but not the swelling time depended on the osmolarity. A pronounced RVD was observed after a hypotonic shock with 40% H _2_ O ( Fig. 2H ). Contrary to some other mammalian cell types, 58 RVD in platelets did not depend on the actin cytoskeleton. 74 We found that platelet activation by thrombin, ADP, or collagen significantly reduced RVD of individual platelets ( Fig. 3 ), like RVD inhibition with high K ^+^ .

The platelet peak volume *V*_p_ followed the Boyle–van't Hoff relationship for different osmotic conditions, from which we quantitatively determined the nonosmotic fraction of individual platelets as 0.33 on average ( Fig. 4 ), in good agreement with the literature. In contrast to collagen-treated platelets, thrombin- and ADP-treated platelets showed a significant decrease (42 and 55%, respectively) in the nonosmotic fraction and volume ( Fig. 4C, D ), which has not been shown on a single-cell level before.

In conclusion, this work shows that HS-SICM is a versatile tool for resolving rapid morphological changes and volume dynamics of adherent living platelets. To further investigate and correlate vesicle fusion with our observations, future studies could utilize capacitance measurements to provide insights into potential volume changes induced by thrombin or ADP during vesicle fusion and the associated membrane surface changes. 71 Future studies could employ SICM for measuring mechanical 75 and electrical 76 properties of adherent platelets, making SICM a valuable addition to well-established technologies for measurement of suspended platelets. Such studies could provide new insights into the links between platelet volume regulation mechanisms, mechanics, membrane function, and compartmentalization in health and disease.

## Notes

**What is known about this topic?**

- Platelets show a regulatory volume decrease after hypotonic shock.
- Measurements of platelet volume regulation have been performed qualitatively on large platelet ensembles.

**What does this paper add?**

- Quantitative, time-resolved measurement of the volume regulation after hypotonic shock of individual platelets using HS-SICM.
- The regulatory volume decrease is suppressed in activated platelets.
- Determination of the nonosmotic fraction of individual platelets via the Boyle–van't Hoff relationship.
- Thrombin- and ADP-activated platelets show a decreased nonosmotic fraction.
