# Supplementary material for: Volume Regulation and Nonosmotic Volume of Individual Human Platelets Quantified by High-Speed Scanning Ion Conductance Microscopy
Source: Thromb Haemost. 2024 Aug 29;125(4):340–51. doi: 10.1055/a-2378-9088 (PMC11961229; doi:10.1055/a-2378-9088)
Supplement: Supplementary file 4 — Supplementary Material [file 10-1055-a-2378-9088-s23120573.pdf]

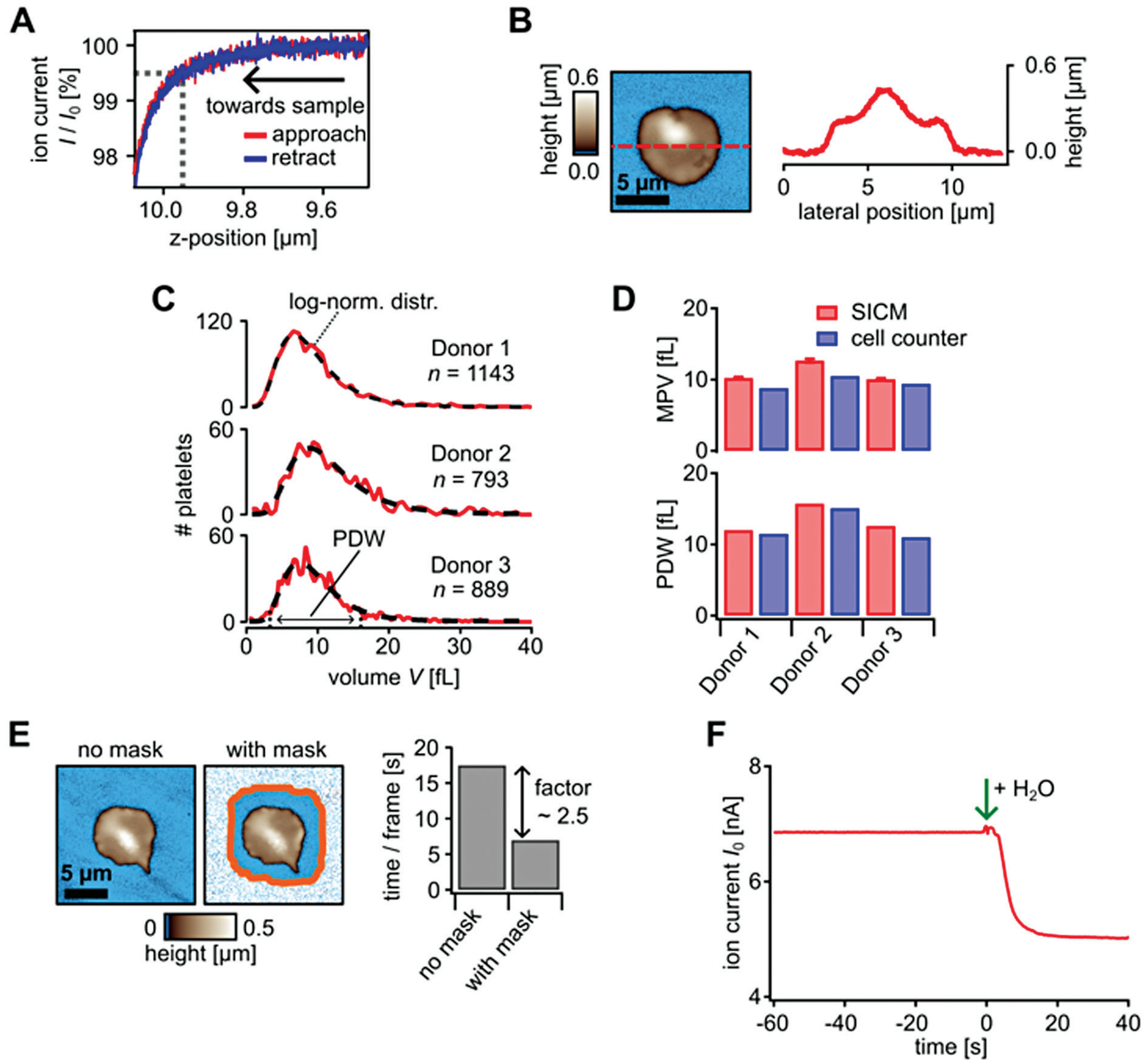

Supplementary Fig. S1 (**A**) Ion current as a function of the *z*-position. The current decreases with a decreasing pipette–sample distance. The dashed lines indicate the current drop at 99.5% of the saturation current I_0 with the corresponding *z*-position. (**B**) High-resolution topography image of a platelet and its height profile along the dashed line. (**C**) HS-SICM platelet volume distributions for three individual donors and log-normal fit (black dashed curves). (**D**) Comparison of MPV and PDW measurements using HS-SICM and a cell counter (gold standard). The MPV and the PDW calculated from the HS-SICM data on washed platelets are in good agreement (within 10–25% deviation) with the values from the cell counter (data acquired from whole blood). (**E**) Imaging speed improvement (factor 2.5) by reducing the number of pixels on the substrate. (**F**) Saturation ion current I_0 as a function of time before and after the exchange of the isotonic buffer solution with a hypotonic buffer solution with 40% H_2O at $t = 0$ s. HS-SICM, high-speed scanning ion conductance microscopy; MPV, mean platelet volume; PDW, platelet distribution width.

 © 2024. The Author(s).

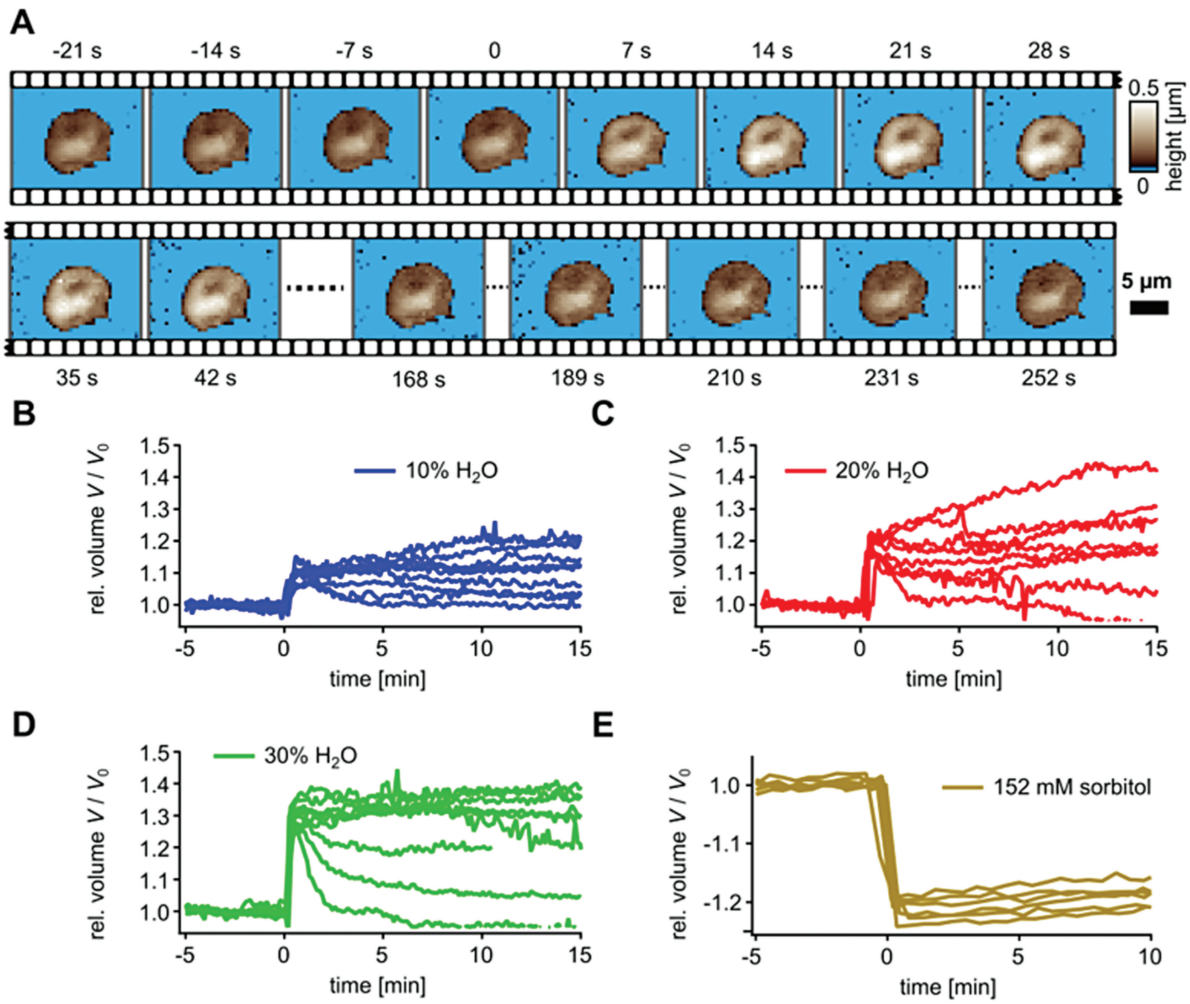

Supplementary Fig. S2 (**A**) Platelet topography image series of Fig. 1C showing more frames at the used frame rate of 7 seconds per frame. A hypotonic shock with 40% H_2O was induced at time $t = 0$. (**B–E**) Volume vs. time curves of individual platelets at different osmotic shock conditions (B: 10% H_2O; C: 20% H_2O; D: 30% H_2O; E: 152 mM D-sorbitol).

 © 2024. The Author(s).

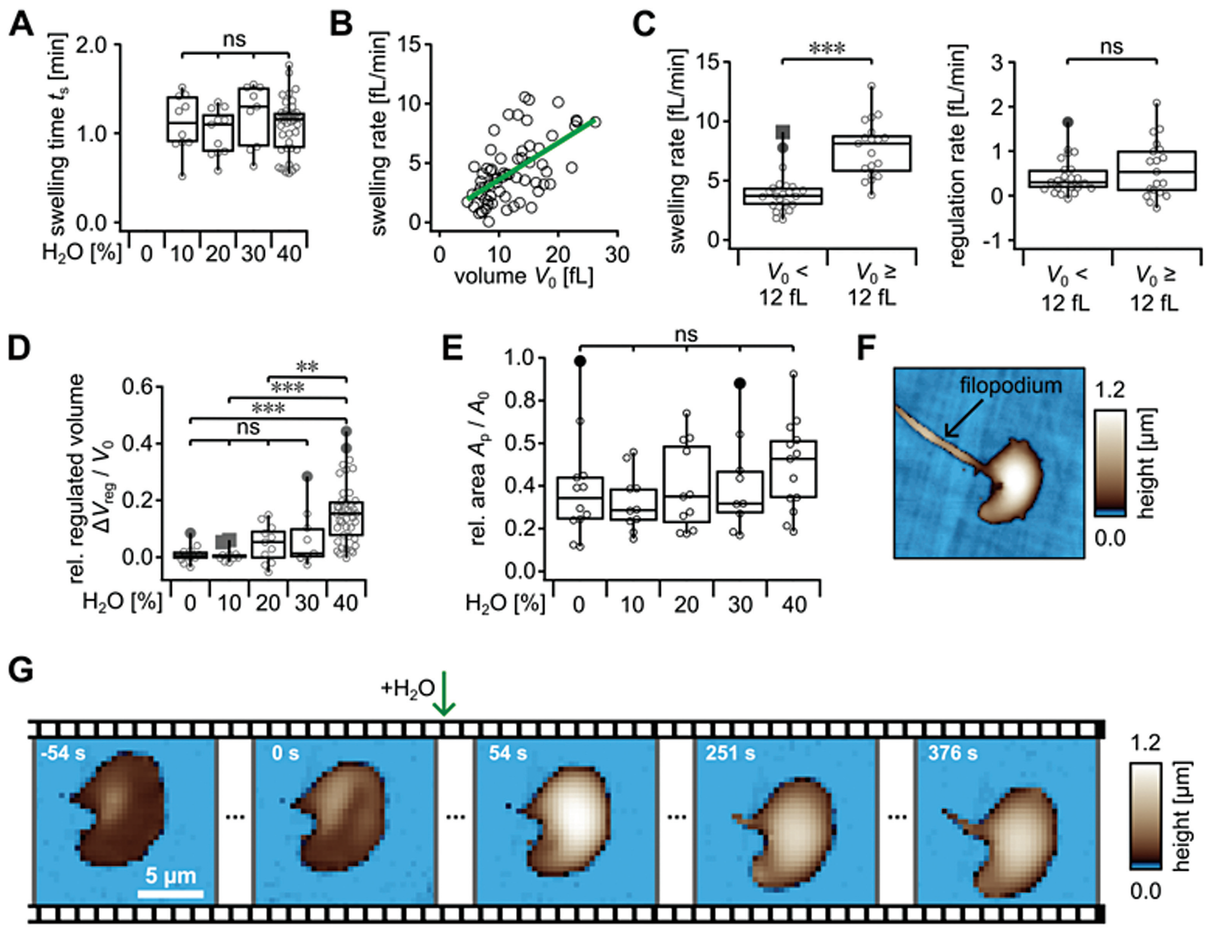

Supplementary Fig. S3 (**A**) Swelling time t_s for a hypotonic shock with different osmolarities. (**B**) Swelling rate as a function of the initial volume V_0, showing a linear correlation. (**C**) Swelling rate (left) and regulation rate (right) of small ($V_0 < 12$ fL) and large ($V_0 \geq 12$ fL) platelets, at a hypotonic shock with 40% H_2O. (**D**) Relative regulated volume $\Delta V_{reg}/V_0$ for different H_2O percentages. (**E**) Relative area (A_p/A_0) at $t = t_s$ for different H_2O percentages, with initial area A_0. (**F**) Platelet topography image after hypotonic shock with 80% H_2O showing the formation of a filopodium (arrow). (**G**) Topography image series of a platelet showing filopodia formation ($t = 376$ s, see ►**Supplementary Video 3** (available in the online version) for the complete image sequence. A hypotonic shock with 80% H_2O was induced at time $t = 0$.

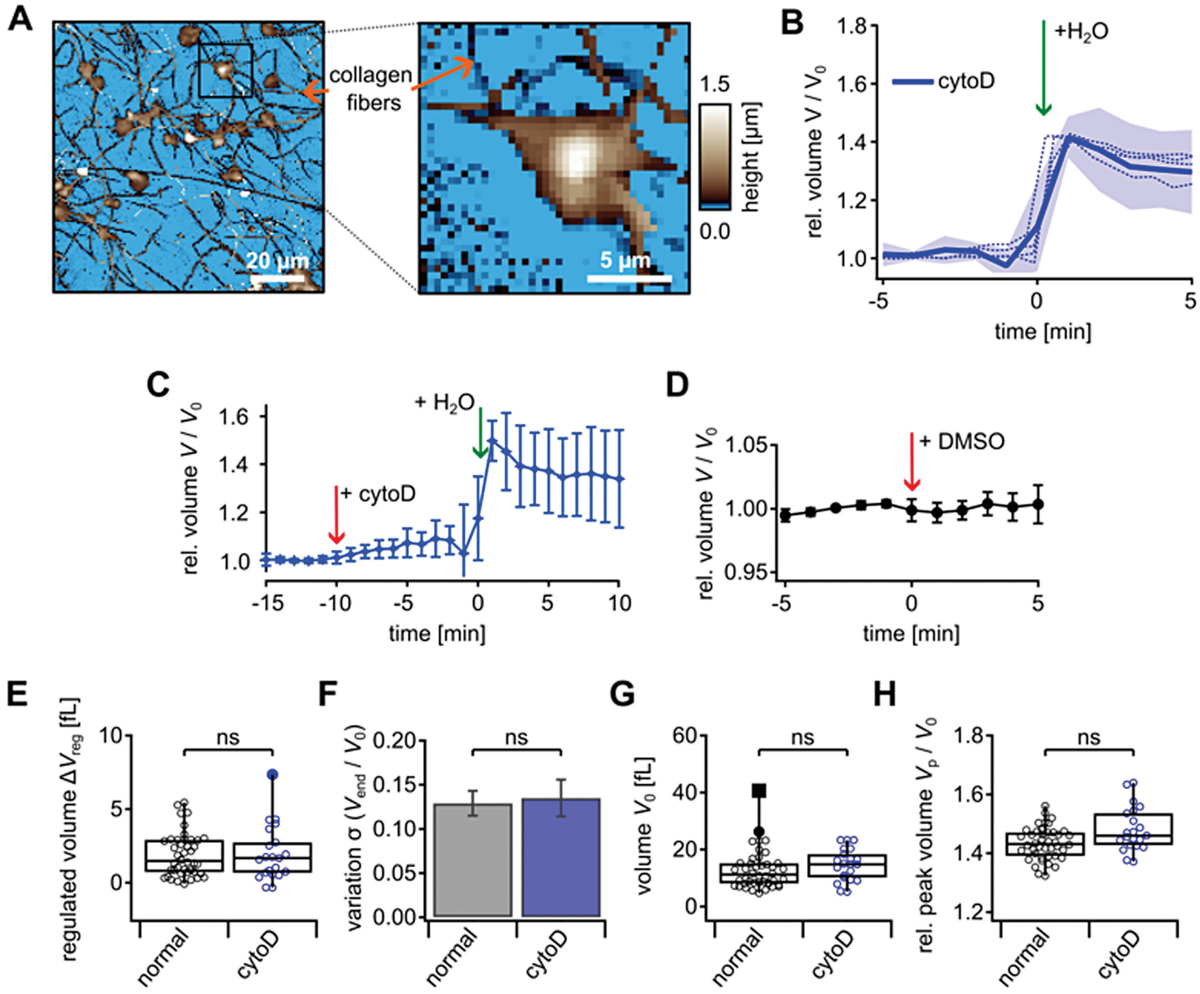

Supplementary Fig. S4 (**A**) Representative SICM topography overview image of platelets on collagen fibers (left) with a zoom-in to a single platelet (right). (**B**) Relative volume vs. time of representative individual platelets (dashed lines) during a hypotonic shock for cytoD-treated platelets. Solid lines and shaded areas represent the mean and standard deviation, respectively, of all measured cytoD-treated platelets. (**C**) Average relative platelet volume (V/V_0) versus time during addition of cytoD at $t = -10$ min and hypotonic shock with 40% H_2O at $t = 0$ min ($N = 21$). (**D**) Average relative platelet volume (V/V_0) versus time during addition of DMSO at $t = 0$ min ($N = 19$), as a control for the cytoD measurements. Error bars denote standard deviation. (**E**) Regulated volume ΔV_{reg} for normal (untreated) and cytoD-treated platelets. (**F**) Variation σ of the relative end volume V_{end}/V_0 for normal and cytoD-treated platelets. Error bars denote the SESD. (**G**) Initial volume V_0 and (**H**) relative peak volume V_p/V_0. Significance in (F) was determined using the F-test. SESD, standard error of the standard deviation; SICM, scanning ion conductance microscopy.

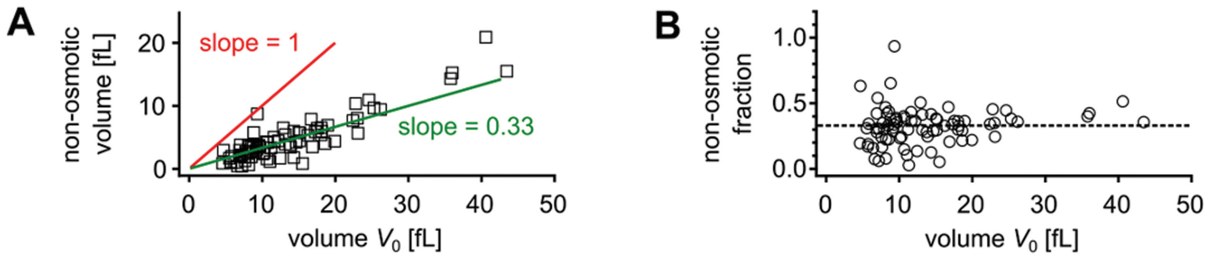

Supplementary Fig. S5 (**A**) Nonosmotic volume (green slope corresponds to a nonosmotic fraction of 0.33 ± 0.01 that is independent of V_0) and (**B**) nonosmotic fraction versus the initial volume V_0 for all measured normal platelets.

 © 2024. The Author(s).
